# Management of hyperglycemia in type 2 diabetes: evidence and uncertainty

**DOI:** 10.1186/1475-2840-12-81

**Published:** 2013-05-30

**Authors:** Katherine Esposito, Sandro Gentile, Riccardo Candido, Alberto De Micheli, Marco Gallo, Gerardo Medea, Antonio Ceriello

**Affiliations:** 1 Department of Clinical and Experimental Medicine, Second University of Naples, Naples, Italy; 2 Diabetes Center, A.S.S. 1 Triestina, Trieste, Italy; 3 Ligurian Health Agency, Genoa, Italy; 4 Oncological Endocrinology, AO Città della Salute e della Scienza-Molinette, Turin, Italy; 5 Italian College of General Practitioners (Società Italiana di Medicina Generale), Florence, Italy; 6 Institut d’Investigations Biomèdiques August Pi i Sunyer (IDIBAPS), Barcelona, Spain; 7 Centro de Investigacion Biomèdica en Red de Diabetes y Enfermedades Metabolicas Asociadas (CIBERDEM), Barcelona, Spain

## Abstract

The panoply of treatment algorithms, periodically released to improve guidance, is one mean to face therapeutic uncertainty in pharmacological management of hyperglycemia in type 2 diabetes, especially after metformin failure. Failure of recent guidelines to give advice on the use of specific antidiabetic drugs in patients with co-morbidity may generate further uncertainty, given the frequent association of type 2 diabetes with common comorbidity, including, although not limited to obesity, cardiovascular disease, impaired renal function, and frailty. The Italian Association of Diabetologists (Associazione Medici Diabetologi, AMD) recognized the need to develop personalized treatment plans for people with type 2 diabetes, taking into account the patients' individual profile (phenotype), with the objective of the safest possible glycemic control. As not every subject with type 2 diabetes benefits from intensive glycemic control, flexible regimens of treatment with diabetes drugs (including insulin) are needed for reaching individualized glycemic goals. Whether personalized diabetology will improve the quality healthcare practice of diabetes management is unknown, but specific research has been launched.

## Introduction

In 2011, there were 366 million people with diabetes worldwide, and this is expected to rise to 552 million by 2030, rendering previous estimates very conservative [1]. Diabetes increases the risk of disabling and life-threatening complications from micro and macrovascular disease. Diabetes is one of the first conditions for which disease-specific indicators based on practice guidelines have been used to “score” the quality of care and preventive services. Recent estimates in the US claim that about one half (48.7%) of persons with diabetes still did not meet the targets for glycemic control; only 14.3% met the targets for all three measures of glycemic control (HbA1c <7%), blood pressure (<130/80 mm Hg), or LDL cholesterol (<100 mg/dl) level [2]. This scenario is still far from the objectives of glycemic therapies in type 2 diabetes which, in addition to achieving target HbA1c, ideally should: a) reverse one or more of the underlying pathophysiological processes, b) produce low unwanted effects, c) enhance quality of life of patients, and d) reduce diabetes micro and macrovascular complications, and diabetes-related mortality [3].

### Clinical uncertainty

Uncertainties abound in healthcare. Although clinical uncertainty was supposed to present only rarely management problems for the doctor, it appeared soon as one most important single factor influencing physician behavior [4]. Clinical uncertainty arising from a number of sources has been managed, at least in part, through evidence-based medicine that helps clinicians convert the data of scientific studies into probabilities that can help reduce uncertainty. However, one of the major hurdles is faced by clinicians on daily basis is selecting the best available evidence. Still today, some questions cannot be answered, no matter how one searches the literature, no matter which expert one consult [5]. Unavoidable clinical uncertainty may have the potential to contribute to clinical inertia, defined as the failure of health care providers to initiate or intensify therapy when indicated [6]. Uncertainty about effectiveness is the oldest source of clinical uncertainty, and is not limited to diabetes: it pushes physicians to rely on inductive reasoning to draw conclusions about the effectiveness and feasibility of application of trial data (mean group data) to individual patients in the real world.

### Management of hyperglycemia in type 2 diabetes

Uncertainties also abound in pharmacological management of hyperglycemia in type 2 diabetes. Sources of uncertainties include, but are not limited to, the panoply of glycemic (HbA1c) targets, the ideal sequence of drugs after metformin failure, the complexity of drug therapy, the possible harms of anti-hyperglycemic drugs, the outcomes of treatment (surrogate versus clinical), and the hierarchy of risk factors to treat in order to prevent the vascular complications. The rising number of diabetes medications available today (more tomorrow) makes it hard, if not impossible, to explore all possible combinations and sequences of combinations that could be recommended. As a corollary, treatment algorithms cannot be truly evidence-based because of a lack of studies comparing all available treatment combination options.

Another source of uncertainty was recently addressed by Tschöpe et al. [7], who stressed the failure of recent guidelines to give advice on the use of specific antidiabetic drugs in patients with co-morbidity. As the patient with type 2 diabetes represents the paradigm of associated co-morbidities (overweight or obesity, dyslipidemia, hypertension, cardiovascular disease, impaired renal function), the expert opinion released by Tschöpe and colleagues [7] seems well-timed from a clinical practice perspective. The evidence presented in support of their expert opinion was the best available; however, the divarication between the world of randomized controlled trials (RCTs) and the real world of the average type 2 diabetic patient remains problematic. The otherwise very complete Figure accompanying the article is a long list of drugs recommended, contraindicated or neutral, among which clinicians may pick up the most appropriate drug for that particular patient, based on their own clinical judgment (a mixture of clinical experience, knowledge and skill).

**Figure 1:**
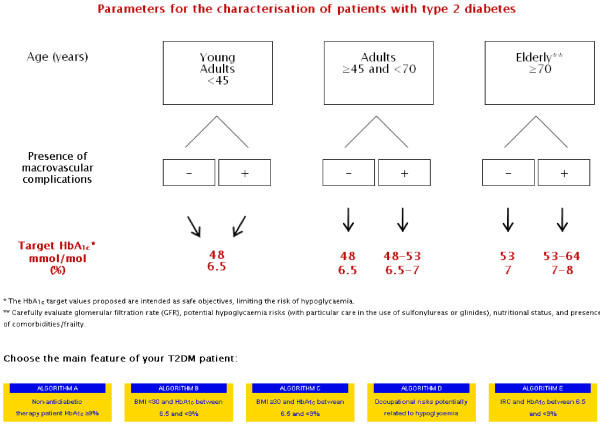
**Therapeutic algorithm developed by the Italian Association of Diabetologists** (**AMD**) **for some of the most common type 2 diabetes phenotypes.Starting page.** The algorithm can be found at http://www.aemmedi.it/algoritmi_it_2013. Accessed 1 June 2013.

Recently, the Italian Association of Diabetologists (Associazione Medici Diabetologi, AMD) recognized the need to develop personalized treatment plans for people with type 2 diabetes, taking into account the patient s individual profile (phenotype), with the objective of the safest possible glycometabolic control. Accordingly, tailored therapeutic algorithms have been developed for some of the most common type 2 diabetes phenotypes [8], as reported, for example, in Figure 1. These algorithms are available in English online as a browser operated interactive version [9]. The reader can quickly locate the subject of interest according to his or her clinical features, and also easily follow a step-by-step suggested additive therapeutic pathway. Online publication facilitates timely updating of the recommendations, ensuring that all healthcare professionals have the latest version of the algorithms readily available at their office. Hopefully, this would also allow for a better adherence to drug therapy. A retrospective analysis of pharmacy claims in a database of more than 64 million members enrolled in 100 health plans assessed persistence and adherence to drug therapy in 6 chronic conditions including type 2 diabetes: 12-month adherence rate for oral antidiabetic drugs was 72%, and as low as 40% at 2 year [10].

### Set the HbA1c target, first

The choice of the HbA1c target was a neglected area in the expert opinion [7]. This is not without practical consequence, as the preliminary individualization of the appropriate HbA1c target for the particular patient physician faces in the real word can remove some of the inconsistencies and gaps with respect to the selection of antidiabetic drug treatment in patients with co-morbid disease conditions. Take, for example, the presence of macrovascular complications in a middle-aged (arbitrarily defined as >45 years) or elderly (>70 years) patient: in these situations, the target would necessarily be translated to upper levels (from 7 to 8% HbA1c), which would result in a lesser aggressive therapeutic pattern, and hence lower side-effects (drug-related). The premise that not every subject with type 2 diabetes benefits from intensive glycemic control, consequently leads setting up flexible regimens of treatment with diabetes drugs (including insulin) and providing individualized glycemic goals and ongoing professional support [11].

### Obesity

Obesity is a frequent co-morbidity of type 2 diabetes. The World Health Organization estimates that obesity accounts for 44% of the global diabetes burden, 23% of the ischemic heart disease burden and 7–41% of the burden of certain cancers globally [12]. Even modest weight loss (5–10% of body weight) has been shown to improve metabolic function and reduce the risk of comorbidities in obese individuals [13]. As a corollary, diabetes drugs that are associated with an unwanted effect of weight gain should be avoided or used wisely. For obese, metformin-treated patients who fail to achieve the individualized target for HbA1c, second-line treatment choices in order of weight benefit would be: GLP-1 agonists, DPP-4 inhibitors, acarbose, bile acid sequestrants, and amylin analogs [7]. In a meta-analysis of GLP-1-based therapies, GLP-1 agonists were associated with significant body-weight reductions from baseline ranging from 2 to 2.4 kg [14]. The incretin effect of GLP-1 is impaired in obesity, and this may contribute to the hyperglycemia, increased appetite and faster gastric emptying that often accompany obesity. In the AMD portfolio, GLP1-agonists are suggested as the first choice for those obese diabetic patients failing metformin, with the only exception of those with isolated postprandial hyperglycemia, where acarbose may also be considered.

### Is personalized diabetology the answer?

Usually accompanying pharmacogenetics, genomics and cancer medicine, personalized medicine is a medical model emphasizing the customization of healthcare, with all decisions and practices being tailored to individual patients in whatever ways possible. Development strategies that administer therapies to unselected populations will perhaps become a strategy of the past. However, physician feeling and conviction about the willingness to reach the HbA1c target (now tailored on the patient) remains paramount to reduce unnecessary therapeutic inertia. Suggested choices within the algorithm represent the best compromise among the scientific evidence of efficacy and safety coming from RCTs, and the translation in the real word of type 2 diabetes.

For the pragmatic physician, however, the evidence that intensive glycemic control may give benefit on microvascular complications of type 2 diabetes [15] may be just enough to accept and propose it for most diabetic patients. This, however, must be tempered with the evidence that intensive glycemic control increases the risk of severe hypoglycemia [16,17]. Fourteen clinical trials that randomized 28 614 participants with type 2 diabetes (15 269 to intensive control and 13 345 to conventional control) were included in a meta-analysis that considered the effects of intensive glycemic control irrespective of differences among trials in individual targets or achieved glycemic control [17].

Personalized diabetology [18] has the potential to improve the quality healthcare practice of diabetes management, but specific research is needed. Personalized diabetology should also take advantage from technological advances and interventions involving mobile applications that may have a positive impact on diabetes self-management [19]. A recent controlled study suggests that a nurse-led online disease management program can achieve greater decreases in A1C at 6 months, although the differences were not sustained at 12 months [20]. It is not by chance that AMD has launched an interventional, national-planned, trial specifically devoted to test the hypothesis whether a strict adherence to the personalized treatment plans would result in better outcomes for type 2 diabetic patients.

## Competing interests

The Authors take full responsibility for the content of this article. Katherine Esposito and Antonio Ceriello serve as guarantors. KE, SG, RC, ADM, MG, GM, and AC received consultancy fees, attended advisory boards or have held lectures for a number of pharmaceutical companies producing antidiabetic drugs.

## Authors’ contributions

The present manuscript has been developed over the course of a virtual meeting in which all authors discussed the data on antidiabetic pharmacotherapy in type 2 diabetic patients. KE wrote the manuscript. All authors revised the article for important intellectual content. All authors read and approved the final manuscript.
